# Oxidative Stress and Inflammatory Biomarkers for the Prediction of Severity and ICU Admission in Unselected Patients Hospitalized with COVID-19

**DOI:** 10.3390/ijms22147462

**Published:** 2021-07-12

**Authors:** Morgane Ducastel, Camille Chenevier-Gobeaux, Yassine Ballaa, Jean-François Meritet, Michel Brack, Nicolas Chapuis, Frédéric Pene, Nicolas Carlier, Tali-Anne Szwebel, Nicolas Roche, Benjamin Terrier, Didier Borderie

**Affiliations:** 1 Department of Automated Biological Diagnostic, Cochin Hospital, APHP-Centre Université de Paris, 75014 Paris, France; morgane.ducastel@aphp.fr (M.D.); yaballaa2@gmail.com (Y.B.); didier.borderie@aphp.fr (D.B.); 2 Department of Virology, Cochin Hospital, APHP-Centre Université de Paris, 75014 Paris, France; jean-francois.meritet@aphp.fr; 3 The Oxidative Stress College Paris, 75007 Paris, France; brackmichel@free.fr; 4 Department of Haematology, Cochin Hospital, APHP-Centre Université de Paris, 75014 Paris, France; nicolas.chapuis@aphp.fr; 5 Medical Intensive Care Unit, Cochin Hospital, APHP-Centre Université de Paris, 75014 Paris, France; frederic.pene@aphp.fr; 6 INSERM U1016, CNRS UMR 8104, Université de Paris, 75014 Paris, France; 7 Department of Pulmonology, Cochin Hospital, APHP-Centre Université de Paris, 75014 Paris, France; nicolas.carlier@aphp.fr (N.C.); nicolas.roche@aphp.fr (N.R.); 8 Institut Cochin, UMR 1016, Université de Paris, 75014 Paris, France; 9 Department of Internal Medicine, Cochin Hospital, APHP-Centre Université de Paris, 75014 Paris, France; tali-anne.szwebel@aphp.fr (T.-A.S.); benjamin.terrier@aphp.fr (B.T.); 10 Centre de Référence Maladies Auto-Immunes et Maladies Systémiques Rares d’Ile-de-France, Université de Paris, 75014 Paris, France; 11 PARCC, INSERM U970, Université de Paris, 75006 Paris, France; 12 INSERM UMRs 1124, Environmental Toxicity, Therapeutic Targets, Cellular Signaling and Biomarkers, Université de Paris, 75006 Paris, France

**Keywords:** biomarker, COVID-19, thiol, oxidative stress, inflammation, outcome, SARS-CoV-2

## Abstract

Objective: We aimed to investigate the prognostic performances of oxidative stress (OS), inflammatory and cell activation biomarkers measured at admission in COVID-19 patients. Design: retrospective monocentric study. Setting: patients with suspected SARS-CoV-2 infection (COVID-19) admitted to the hospital. Patients: One hundred and sixty documented and unselected COVID-19-patients. Disease severity (from mild to critical) was scored according to NIH’s classification. Interventions: none. Measurements and main results: We measured OS biomarkers (thiol, advanced oxidation protein products (AOPP), ischemia-modified albumin (IMA)), inflammation biomarkers (interleukin-6 (IL-6), presepsin) and cellular activation biomarkers (calprotectin) in plasma at admission. Thiol concentrations decreased while IMA, IL-6, calprotectin and PSEP increased with disease severity in COVID-19 patients and were associated with increased O_2_ needs and ICU admission. The best area under the receiver-operating-characteristics curve (AUC) for the prediction of ICU admission was for thiol (AUC = 0.762). A thiol concentration <154 µmol/L was predictive for ICU admission (79.7% sensitivity, 64.6% specificity, 58.8% positive predictive value, 78.9% negative predictive value). In a stepwise logistic regression, we found that being overweight, having dyspnoea, and thiol and IL-6 plasmatic concentrations were independently associated with ICU admission. In contrast, calprotectin was the best biomarker to predict mortality (AUC = 0.792), with an optimal threshold at 24.1 mg/L (94.1% sensitivity, 64.9% specificity, 97.1% positive predictive value and 98.9% negative predictive value), and survival curves indicated that high IL-6 and calprotectin concentrations were associated with a significantly increased risk of mortality. Conclusions: Thiol measurement at admission is a promising tool to predict ICU admission in COVID-19-patients, whereas IL-6 and calprotectin measurements effectively predict mortality.

## 1. Introduction

Coronavirus Disease 19 (COVID-19) is caused by the new emerging severe acute respiratory syndrome coronavirus 2 (SARS-CoV-2), responsible for more than 124,000,000 confirmed cases worldwide and 2,700,000 deaths [1]. Disease severity ranges from asymptomatic to mild-to-moderate forms, and more rarely severe acute respiratory distress syndrome (ARDS). Many studies have reported the relationship between inflammation and the deterioration of the patient’s condition, and this ‘cytokine storm’ is considered to be a major factor for the development of ARDS and multiple organ dysfunction [2,3,4].

There is a strong association between inflammation and oxidative stress (OS) [5]. Recent studies postulated that OS could be at the crossroad between inflammation and microvascular dysfunction [6,7]. In addition to neutrophil infiltration and excessive release of reactive oxygen species (ROS), viral infections were shown to decrease antioxidant defenses [8]. Deleterious action of ROS on alveolar epithelial and endothelial cell functions could be a major contributor for hypoxic respiratory failure observed in the most severe cases of COVID-19 [9]. SARS-CoV-2 infection can also lead to cellular damage which can initiate toxic and inflammatory stress responses [10]. OS causes molecular modifications such as carbonylation of albumin and formation of advanced oxidation protein products (AOPP), the latter being good indicators of the extent of oxidative damage. Moreover, ROS injury induces structural changes in the N-terminal region of albumin that can be evaluated by the determination of ischemia-modified albumin (IMA). Finally, antioxidant status can be easily evaluated by measuring total plasmatic thiol.

We hypothesized that the oxidative burst, reflected by the imbalance between antioxidant (thiol) and pro-oxidant (AOPP, IMA) biomarkers, could be correlated with COVID-19 severity and help predict ICU admission and mortality. Therefore, we assessed the prognostic performance of OS, inflammatory and cell activation biomarkers in unselected patients with COVID-19.

## 2. Results

### 2.1. Baseline Characteristics of the Studied Population

Baseline characteristics of the study population are presented in Table 1. Briefly, 31 (19.4%) patients were classified as mild cases of COVID-19 (stage 0), 36 (22.5%) patients were moderate cases (stage 1), 36 (22.5%) patients were severe cases (stage 2) and 57 (35.6%) patients were critical cases (stage 3). Critical COVID-19 patients were more frequently men and presented more frequently cardiovascular disease, being overweight, high blood pressure, dyspnea, and fever, in comparison to lower stages. Fifty-one out of 54 ventilated stage 3 patients had orotracheal intubation. Critical patients also presented higher CRP and fibrinogen levels, leucocytes and neutrophils, and showed lower hemoglobin and albumin concentrations. Furthermore, age, increased oxygen needs, ICU admission and length of stay increased across disease severity. Finally, all deceased patients (*n* = 17) were observed amongst critical COVID-19 patients.

### 2.2. Oxidative Stress (OS), Inflammation and Cell-Activation Biomarkers Concentrations across COVID-19 Severity Stages

**Figure 1:**
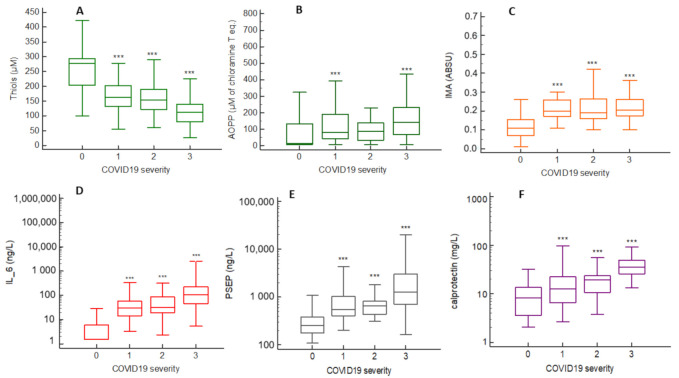
Oxidative stress (OS), inflammation and cell-activation biomarker concentrations across COVID-19 severity stages. (**A**) thiols concentrations; (**B**) AOPP concentrations; (**C**) IMA concentrations; (**D**) IL-6 concentrations; (**E**) presepsin concentrations; (**F**) calprotectin concentrations. ***: *p* < 0.05 vs. severity 0.

We analyzed OS and inflammation/cell-activation biomarkers concentrations across COVID-19 severity stages. For OS biomarkers, we found that thiol concentrations significantly decreased across severity in COVID-19-patients (from 272 (202–295) µmol/L in stage 0 to 112 (79–140) µmol/L in stage 3, *p* < 0.001), while IMA concentrations increased from 0.11 (0.07–0.14) ABSU in stage 0 to 0.21 (0.18–0.26) ABSU in stage 3, *p* < 0.001) (Figure 1A,C). AOPP concentrations were significantly increased only in stages 1 and 3 (80 (44–189) and 140 (67–230) µmol/L of chloramine T eq., respectively) (Figure 1B). Regarding inflammation biomarkers, our results showed that IL-6, PSEP and calprotectin concentrations significantly increased with severity in COVID-19-patients (Figure 1D–F).

### 2.3. Correlations between Biomarkers

We next investigated potential correlations between biomarkers. Coefficients of correlation are presented in Table S1. All the significant correlations observed were weak or moderate, except for the correlation between neutrophils and leukocytes which was strong. Of note is that thiol was significantly correlated with albumin (*r* = 0.615, *p* < 0.001).

### 2.4. Oxidative Stress (OS), Inflammation and Cell-Activation Biomarkers Concentrations According to Increased O_2_ Needs

We analyzed OS and inflammation/cell-activation biomarkers concentrations according to increased O_2_ needs. Thiol concentrations were significantly decreased in patients that presented increased O_2_ needs (130 (91–163) vs. 197 (149–275) µmol/L, *p* < 0.001), while IMA and AOPP concentrations were significantly increased (IMA: 0.20 (0.17–0.27) vs. 0.17 (0.11–0.24) ABSU, *p* < 0.001; AOPP: 112 (47–220) vs. 59 (7–174) µmol/L of chloramine T eq., *p* < 0.001). Inflammation biomarkers (IL-6, PSEP) and cell-activation biomarker (calprotectin) were significantly increased (Table S2).

### 2.5. Prognostic Performances of OS, Inflammation and Cell-Activation Biomarkers for ICU Admission

**Figure 2:**
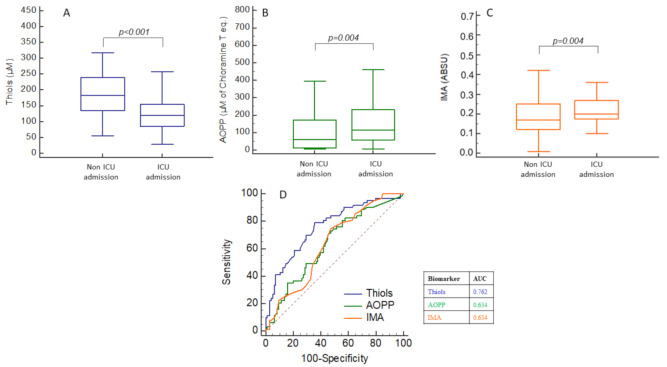

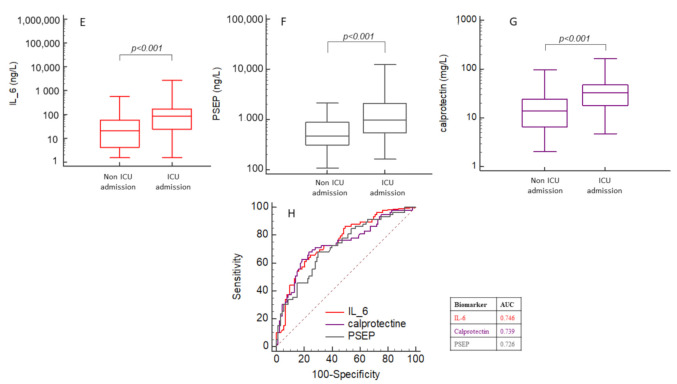
Box-plots and receiver operating characteristics (ROC) curve analysis of oxidative stress (OS), inflammation and cell-activation biomarker concentrations for the prediction of ICU admission. (**A**–**C**), thiols, AOPP and IMA concentrations according to ICU admission; (**D**) thiols AOPP and IMA ROC curves and AUC values; (**E**–**G**) IL-6. presepsin, calprotectin concentrations according to ICU admission; (**H**) IL-6, presepsin, calprotectin ROC curves and AUC values.

We performed receiver operating characteristics (ROC) curve analysis in order to evaluate the prognostic performances of each biomarker regarding ICU admission. Thiol concentrations were significantly decreased in patients admitted to ICU (121 (85–154) vs. 183 (134–239) µmol/L, *p* < 0.001), while all other biomarkers (IMA, AOPP, IL-6, PSEP and calprotectin) were significantly increased (Figure 2A–C,E–G).

The best area under the ROC curve (AUC) for the prediction of ICU admission was for thiol concentrations (AUC = 0.762, *p* < 0.001) (Figure 2D,H). AUC was higher for IMA concentrations (AUC = 0.634, *p* < 0.01 vs. thiol) and AOPP concentrations (AUC = 0.634, *p* < 0.02 vs. thiol). ROC analysis indicated that a thiol concentration <154 µmol/L was predictive of ICU admission with 79.7% sensitivity, 64.6% specificity, 58.8% positive predictive value and 78.9% negative predictive value. ROC analysis of other biomarkers indicated that inflammatory and cell-activation biomarkers had higher AUC than other OS biomarkers such as AOPP and IMA (Table 2). A Venn diagram indicated interactions between low thiol, elevated IL-6 and elevated calprotectin in our study. The proportion of patients with isolated low thiol was higher than the proportion of isolated elevated IL-6 or calprotectin (Figure S1).

We further performed logistic regression to assess variables associated with ICU admission. We included in the model the three best studied biomarkers according to ROC curves: thiol, calprotectin, IL-6. Results are indicated in Table S3. Overweight, dyspnoea, thiol and IL-6 were independently associated to ICU admission (c-statistic = 0.846; Hosmer-Lemeshow test: Chi^2^ = 3.88, *p* = 0.868).

### 2.6. Prognostic Performances of OS, Inflammation and Cell-Activation Biomarkers for Death

**Figure 3:**
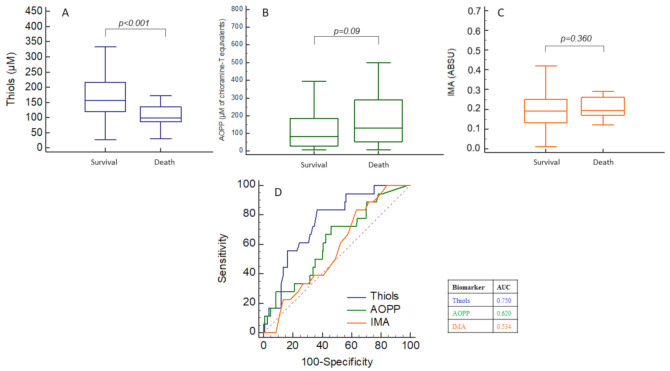

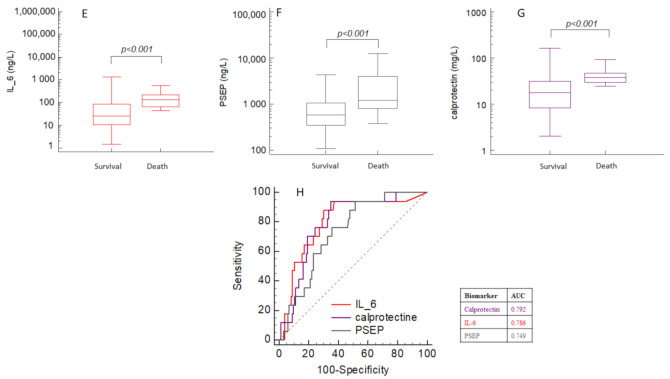
Box-plots and receiver operating characteristics (ROC) curve analysis of oxidative stress (OS), inflammation and cell-activation biomarker concentrations for the prediction of death. (**A**–**C**), thiols, AOPP and IMA concentrations according to death; (**D**), thiols, AOPP and IMA ROC curves and AUC values; (**E**–**G**), IL-6, presepsin, calprotectin concentrations according to death; (**H**) IL-6, presepsin, calprotectin ROC curves and AUC values.

We next analyzed the performance of OS and inflammation/cell-activation biomarker concentrations for the prediction of death. Thiol concentrations were significantly decreased in deceased patients (98 (86–135) vs. 157 (120–217) µmol/L, *p* < 0.001), while other biomarkers (IL-6, PSEP and calprotectin) but not IMA nor AOPP were significantly increased (Figure 3A–C,E–G).

The best AUC for the prediction of death was for calprotectin (AUC = 0.792, *p* < 0.001) (Figure 3D,H). ROC analysis indicated that a calprotectin concentration >24.1 mg/L was predictive for death with 94.1% sensitivity, 64.9% specificity, 97.1% positive predictive value and 98.9% negative predictive value. ROC analysis of OS biomarkers and other inflammatory biomarkers for the prediction of death is shown in Table 2.

We further performed logistic regression to assess variables associated with death. Results are indicated in Table S3. Age and calprotectin were independently associated with death (c-statistic = 0.871; Hosmer-Lemeshow test: Chi^2^ = 11.4, *p* = 0.181).

**Figure 4:**
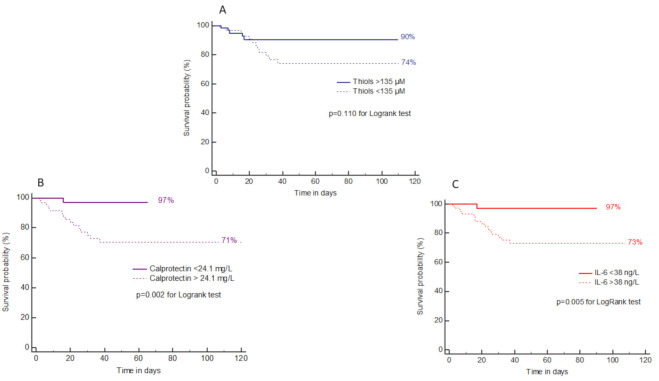
Survival curves according to thiol (**A**), calprotectin (**B**) and IL-6 (**C**) concentrations.

Survival curves indicated that a thiol concentration >135 µmol/L was associated with an increased risk of mortality of 26% (Figure 4A) but without significance, while a calprotectin concentration >24.1 mg/L or an IL-6 concentration >38 ng/L was associated with a significantly increased risk of mortality around 25% (Figure 4B,C).

## 3. Discussion

Several reviews have suggested a role of oxidative stress (OS) in the pathophysiological pathway leading to adverse outcomes associated with COVID-19 [7,9,11,12]. We hypothesized that measuring biomarkers of this pathway may enable early triage and risk stratification of patients with COVID-19 at admission. Indeed, our major finding is that thiol concentrations were significantly decreased with disease severity in COVID-19 patients and were associated with increased O_2_ needs and with ICU admission.

We found that increased levels of pro-oxidative biomarkers (IMA, AOPP) and decreased levels of antioxidant biomarker (total thiol) were related to inflammatory process, as supported by the significant correlation with the different inflammatory biomarkers. Our results may indicate that the antioxidant status in the plasma of patients with COVID-19 depends on inflammatory status. Our study confirmed data from previous studies because we found that IL-6, CRP, PSEP and calprotectin were associated with worse outcomes [13,14,15]. In our study, calprotectin was the best biomarker to predict mortality (AUC = 0.792), with an optimal threshold at 24.1 mg/L (94% sensitivity and 65% specificity), and survival curves indicated that a calprotectin concentration >24.1 mg/L was associated with a significant increase of 25% in risk of mortality. The expression of calprotectin is predominantly restricted to the intracellular compartment of neutrophil granulocytes. In contrast to routinely used inflammatory biomarkers such as C-reactive protein (CRP) and procalcitonin (PCT), calprotectin is released into the bloodstream without de novo protein biosynthesis. This reflects the activation of neutrophils, probably by cytokines, leading to the release of ROS in high amounts. Thus, calprotectin might be correlated to the levels of circulating neutrophil extracellular traps (NETs) [16]. Therefore, circulating calprotectin elevation might be one of the first responses of an organism to an inflammatory disease. Indeed, calprotectin was better than CRP and PCT for admitting COVID-19 patients to the ICU, with an AUC at 0.80 (vs. 0.66 and 0.60, respectively) [17,18]. Considering our results, we may hypothesize that while thiol seems to be the leader in predicting ICU admission, inflammatory biomarkers such as IL-6 and calprotectin may be ‘worsening’ detectors and mortality predictors: these observations are in accordance with what was previously observed [19].

We found that thiol concentrations were significantly decreased in severe COVID-19 patients and in patients admitted to ICU. Thiol was the best biomarker to predict ICU admission (AUC = 0.762), with an optimal threshold at 154 µmol/L (80% sensitivity and 65% specificity). Finally, in a stepwise logistic regression we found that being overweight, having dyspnoea, and thiol and IL-6 were independently associated with ICU admission. Plasma thiol concentration may represent a clinically useful risk stratification tool that provides important insights in hospitalized patients with COVID-19. Our results are in line with those of Kalem et al. that showed a predictive value of total and native thiol in the diagnosis of COVID-19 and in determining disease severity [20]. The single thiol of human serum albumin (HSA) is the most abundant plasma thiol (400–600 µmol/L) and is, for the most part, reduced (75%); the other low molecular weight thiol constitutes only 12–20 µmol/L [21]. Thus, the decrease in total thiol in the plasma mainly reflects the decrease of reduced albumin. Among the 585 amino acid residues in HSA, Methionine-6 and the Cysteine-34 accounted for 40–80% of the total antioxidant activity of HSA [22] and were determined to be the preferred plasma scavenger of reactive oxygen species [23].

Oxidative stress causes other molecular modifications such as carbonylation of HSA and the formation of AOPP. These stable final products are considered to be good indicators of the extent of oxidative damage. It was demonstrated that free radical injury induces structural changes in the N-terminal region of HSA. The ability of HSA to bind transition metal ions such as cobalt, copper and nickel is reduced [24] and can be evaluated by the determination of IMA. We showed the increase of IMA and AOPP concentrations in plasma of patients with COVID-19. IMA and AOPP were inversely correlated with thiol (−0.506, *p* < 0.001; −0.313, *p* < 0.001, respectively). HSA may undergo irreversible oxidation, which impairs its antioxidant property. We observed lower values of HSA in the group of critical patients. These results are consistent with a systematic review study that showed that 75.8% of patients with COVID-19 presented decreased amounts of albumin [25]. In our study, HSA was correlated with thiol in plasma from COVID-19 patients (*r* = 0.615, *p* < 0.001). Therefore, hypoalbuminemia observed in COVID-19 patients may have several explanations. Hypoalbuminemia is a feature of inflammation as confirmed in our study by the inverse relation between albumin and CRP or IL-6, but it might also the consequence of a high clearance of oxidized form of albumin.

The decrease of redox status may occur in the pathophysiological process in COVID-19 patients. Less availability of reduced thiol may play a role in the interaction of viral spike protein of the SARS-CoV-2 and the functional receptor for the SARS coronaviruses, namely the angiotensin-converting enzyme 2 (ACE2). Molecular dynamic simulations showed that the binding affinity was significantly impaired when all the disulphide bonds of both ACE2 and viral spike proteins were reduced to thiol groups and provided a molecular basis for the severity of COVID-19 infection due to oxidative stress [26,27].

## 4. Limitations

Our study may have selection bias as it was a single-center, retrospective study. The sample size of patients hospitalized with COVID-19 in our study was modest compared with published cohort from clinical cases series. However, this retrospective study was performed during the epidemic peak and presented a unique opportunity of collecting homogenous data from the same outbreak. Our findings cannot be extrapolated to patients who do not require hospitalization.

## 5. Material and Methods

### 5.1. Population

From April to May 2020, we collected from Cochin Hospital 278 leftover heparinized plasma samples from patients with suspected SARS-CoV-2 infection (COVID-19). One hundred and sixty patients with confirmed COVID-19 were included (Figure S2). This study was performed according to principles of the Declaration of Helsinki, and was approved by our local ethics committee (Institutional Reviewing board “Comité local d’éthique pour les publications de l’hôpital Cochin”, CLEP Decision N°: AAA-2020-08050), which waived the request for patient consent.

Suspected patients presented signs of respiratory infection such as cough, fever, dyspnoea, myalgia, fatigue and/or diarrhoea. Patients’ clinical and biological data were collected as follows: symptoms, comorbidities, blood routine biomarkers (albumin, CRP, fibrinogen, leukocytes count, neutrophils count) and COVID-19 status according to RT-PCR results performed at admission and/or seroconversion.

Patients were classified according to severity from mild (stage 0) to critical (stage 3) adapted from the NIH COVID-19 Treatment Guidelines [28]:

Mild cases had symptoms without dyspnoea or abnormal imaging (COVID-19 stage 0).

Moderate cases showed evidence of lower respiratory disease with SpO2 > 94% (COVID-19 stage 1).

Severe cases had SpO2 < 94% or respiratory rate > 30 or lung infiltrates higher than 50% on computed tomography (COVID-19 stage 2)

Critical cases presented acute respiratory distress syndrome or septic shock (COVID-19 stage 3).

### 5.2. Biomarkers Measurements

A panel of consolidated biomarkers was assessed routinely at admission, including C-reactive protein (CRP), albumin, fibrinogen, leukocytes and neutrophils count.

Samples taken from routine check-ups on a median of 1 (range 0–4) day after the patient’s admission were collected and left-over heparinized plasma frozen at −80 °C for subsequent measurement of OS (thiol, AOPP and IMA), inflammation (interleukin-6, presepsin) and neutrophil activation (calprotectin) biomarkers.

Determination of thiol levels was based on the thiol/disulfid reaction of thiol and Ellman’s reagent (5,5′-dithiolbis(2-nitrobenzoic acid), DTNB) (Sigma Aldrich, Saint-Quantin Fallavier, France) [29]. Advanced Oxidation Protein Products (AOPP) were measured using the chloramine-T method [30]. Ischemia-modified albumin (IMA) concentrations were measured using the albumin cobalt binding test [31].

Interleukin-6 (IL-6) concentrations were measured using the IL-6 ECLIA assay on a cobas E801 module integrated on a cobas^®^8000 analyser (Roche Diagnostics Meylan, France). Presepsin (PSEP) was measured using the ST AIA-PACK PRESEPSIN and immunoenzymometric assay on a AIA360 analyser (Tosoh Bioscience, Tosoh Europe N.V., Tessenderlo, Belgium). Calprotectin concentrations were measured using the turbidimetric assay (Bühlmann, Mulhouse, France) adapted on a cobas c501 analyser (Roche Diagnostics Meylan, France) [32].

Physicians in charge of the patients were blinded to the results of biomarkers, and biologists were blinded to the diagnosis suspected by physicians.

### 5.3. Statistical Analysis

Continuous variables were presented as median (25th–75th percentile) and categorical variables as numbers (percentages). Continuous variables were compared with the Mann-Whitney U test and categorical variables using the Pearson chi-square test. Receiver-operator characteristic (ROC) curves were constructed to assess the sensitivity and specificity, positive (PPV) and negative predictive value (NPV) (all with their 95% confidence interval [95% CI]) throughout the concentrations of biomarkers, to compare the accuracy of these biomarkers for risk-stratification (ICU-admission or death). Comparison of areas under ROC curves was performed. The normality of the distribution was tested with the Kolmogorov-Smirnov test for all investigated biomarkers. When the distribution was not normal, a log-transformation was performed. Log-transformed values were therefore used in subsequent analysis (correlation and logistic regression). Correlation between biomarkers was assessed using Spearman rank correlation in order to determine the multicollinearity between two variables. Collinearity provide the possibility of including only one of the two variables in the same regression model [33]. A stepwise logistic regression was performed to assess variables associated with ICU-admission and mortality. Biomarkers were selected in the model according to their pathophysiological category (oxidative stress, inflammation, neutrophilic activation), and variables of the same category but less performant in univariate analysis were excluded to avoid redundancy. Only variables with *p* values < 0.20 in the univariate analysis were included in the regression analysis. The discriminate power of the logistic regression was evaluated by the c-statistic (concordance index) and the goodness of fit of the model using the Hosmer-Lemeshow test. We also performed survival analysis building Kaplan-Meyer curves according to biomarker value. Survival time was defined as the time from hospital admission to the date of discharge or death. A *p* value of <0.05 was considered significant. Statistical analysis was performed using MedCalc (MedCalc Software, Mariakerke, Belgium).

## 6. Conclusions

In hospitalized COVID-19-patients, low thiol plasma concentrations were correlated with the severity of the disease and demonstrated to be a promising tool to predict ICU admission, whereas IL-6 and calprotectin measurements effectively predicted mortality.

## Figures and Tables

**Table 1:** Baseline characteristics of the studied population.

Baseline Characteristics	COVID-19 Patients According to Severity								
	Mild (Stage 0)		Moderate (Stage 1)		Severe (Stage 2)		Critical (Stage 3)		
	N	Value	N	Value	N	Value	N	Value	*p*
Men—n (%)	31	11 (35.5)	36	20 (55.6)	36	18 (50)	57	43 (75.4)	<0.001
Age—years (IQR)	31	47.4 (32.4–57.1)	36	63.9 (49.1–75.8)	36	64.7 (58.5–74.9)	57	61.9 (51.2–73.9)	<0.001
Smoking—n (%)	31	4 (12.9)	36	9 (25.0)	36	9 (25.0)	57	12 (21.1)	0.542
Cardiovascular Disease—n (%)	30	6 (20)	34	19 (55.9)	36	20 (55.6)	53	30 (56.6)	0.006
Overweight/obesity—n (%)	31	6 (19.4)	36	17 (47.2)	36	17 (47.2)	57	42 (73.7)	<0.001
Hypertension—n (%)	30	3 (10)	36	16 (44.4)	36	16 (44.4)	54	26 (48.1)	0.003
Diabetes—n (%)	30	1 (3.3)	36	10 (27.8)	36	13 (36.1)	54	13 (24.1)	0.068
Chronic kidney Disease—n (%)	30	1 (3.3)	36	7 (19.4)	36	1 (2.8)	53	7 (13.2)	0.568
Chronic Resp Failure—n (%)	30	2 (6.7)	36	1 (2.8)	36	2 (5.6)	54	3 (5.6)	0.961
Systemic autoimmune Disease—n (%)	30	1 (3.3)	35	4 (11.4)	36	1 (2.8)	54	3 (5.6)	0.851
**Symptoms and Clinic at Admission:**									
Temperature > 38 °C—n (%)	31	9 (29.0)	36	30 (83.3)	35	30 (85.7)	52	41 (78.8)	<0.001
Cough—n (%)	31	19 (61.3)	36	24 (66.7)	36	23 (63.9)	54	31 (57.4)	0.580
Dyspnea—n (%)	31	9 (29.0)	36	17 (47.2)	36	25 (69.4)	55	44 (80)	<0.001
Myalgias—n (%)	31	14 (45.2)	36	11 (30.6)	36	13 (36.1)	55	11 (20)	0.026
Fatigue—n (%)	31	3 (9.7)	36	21 (58.3)	36	19 (52.8)	55	21 (38.2)	0.103
Diarrhea—n (%)	31	10 (32.3)	36	10 (27.8)	36	10 (27.8)	55	10 (18.2)	0.142
Oxygenation—n (%)	31	1 (3.2)	36	28 (77.8)	36	36 (100)	57	57 (100)	<0.001
Admission flow (L/min)	1	ND	26	2.0 (1.0–2.0)	35	4.0 (2.6–5.5)	45	5 (3.0–15.0)	<0.001
Extension at TDM: <10% 10–25% 25–50% 50–75% >75%	31 3 3 3 3 3	5 (16.1) 2 (66.7) 0 (0) 1 (33.3) 0 (0) 0 (0)	36 29 29 29 29 29	33 (91.7) 4 (13.8) 9 (31.0) 13 (44.8) 3 (10.3) 0 (0)	36 33 33 33 33 33	35 (97.2) 0 (0) 6 (18.2) 14 (42.4) 10 (30.3) 3 (9.1)	51 31 31 31 31 31	44 (86.3) 0 (0) 4 (12.9) 12 (38.7) 9 (29.0) 6 (19.4)	<0.001 <0.001
O_2_ needs majoration —n (%)	31	0 (0)	36	10 (28)	36	24 (67)	57	55 (96)	<0.001
Ventilation—n (%)	31	0 (0)	36	1 (2.8)	36	13 (36.1)	57	54 (94.7)	<0.001
ICU Admission—n (%)	31	1 (3.2)	36	1 (2.8)	36	10 (27.8)	57	52 (91.2)	<0.001
Length of stay—n (%)	31	0 (0–0)	36	7 (4–10)	36	10 (7–19)	56	26 (16–50)	<0.001
Death—n (%)	31	0 (0)	36	0 (0)	36	0 (0)	57	17 (29.8)	<0.001
**Blood Routine Biomarkers at admission, when performed**									
Albumin—g/L (IQR)	6	38 (30–42)	28	34.5 (31–37)	28	32.5 (29–36)	49	25 (22–30)	<0.001
CRP—mg/L (IQR)	14	1.8 (1.0–23.0)	34	74.9 (28.8–118.3)	34	98.4 (45.7–166.7)	42	162 (48.6–234)	<0.001
Fibrinogen—g/L (IQR)	25	3.3 (2.7–3.8)	25	6.2 (4.5–7.3)	32	6.9 (5.5–7.9)	47	6.6 (4.9–8.0)	<0.001
Leukocytes—G/L (IQR)	30	4.69 (4.04–6.10)	35	5.08 (3.94–7.46)	36	6.61 (4.75–9.23)	57	9.35 (7.64–12.4)	<0.001
Neutrophils—G/L (IQR) Neutrophils/Lymphocytes	30 30	2.75 (2.30–3.62) 2.0 (1.5–2.3)	34 34	3.65 (2.46–5.24) 3.7 (2.3–5.7)	36 36	4.66 (3.39–6.87) 4.5 (3.4–9.2)	56 56	7.55 (5.88–11.0) 8.5 (4.9–14.4)	<0.001 <0.001

Values are expressed in numbers (%) or in median (IQR).

**Table 2:** AUC, optimal threshold, sensibility and specificity obtained by the ROC curves for prediction of ICU admission and mortality.

Biomarker	AUC	p	Optimal Threshold (*)	Sensibility in % [95% CI]	Specificity in % [95% CI]	Positive Predictive Value in % [95% CI]	Negative Predictive Value in % [95% CI]
**For prediction of ICU admission**							
Thiols	0.762	<0.001	<154 µmol/L	79.7 [67.8–88.7]	64.6 [54.2–74.1]	58.8 [47.2–69.5]	78.9 [68.1–86.9]
IL-6	0.746	<0.001	>60.9 ng/L	59.4 [46.4–71.5]	78.1 [68.5–85.9]	62.3 [48.9–74.1]	73.7 [63.7–81.8]
Calprotectin	0.739	<0.001	>28.1 mg/L	62.7 [49.1–75.0]	81.7 [72.4–89.0]	63.5 [50.4–75.0]	78.7 [68.5–86.4]
PSEP	0.726	<0.001	>721 ng/L	68.3 [55.3–79.4]	70.8 [60.7–79.7]	60.6 [48.3–71.8]	77.3 [66.9–85.3]
AOPP	0.634	0.003	>70 µmol/L	73.0 [60.3–83.4]	52.1 [41.6–62.4]	50.0 [39.5–60.5]	74.6 [62.2–84.1]
IMA	0.634	0.002	>0.17 ABSU	74.6 [62.1–84.7]	52.6 [42.1–63.0]	51.1 [40.5–61.6]	75.8 [63.4–85.1]
**For prediction of mortality**							
Calprotectin	0.792	<0.001	>24.1 mg/L	94.1 [71.3–99.9]	64.9 [56.2–73.0]	97.1 [88.4–99.5]	98.9 [93.0–99.9]
IL-6	0.786	<0.001	>37.6 ng/L	94.4 [72.7–99.9]	59.6 [51.0–67.7]	23.0 [14.3–34.5]	98.8 [92.7–99.9]
Thiols	0.750	<0.001	<135 µmol/L	83.3 [58.6–96.4]	63.1 [54.6–71.1]	21.0 [12.1–33.6]	94.8 [87.8–98.1]
PSEP	0.749	<0.001	>545 ng/L	94.4 [72.7–99.9]	49.3 [40.7–57.9]	19.3 [12.0–29.4]	98.6 [91.3–99.9]
AOPP	0.620	0.027	>88 µmol/L	72.2 [46.5–90.3]	53.2 [45.–62.0]	16.7 [9.5–27.2]	93.8 [85.4–97.7]
IMA	0.534	0.599	/	/	/	/	/

NB. Biomarkers are classified from to the highest AUC (upper line) to the lowest (lower line). (*) according to ROC analysis.

## Data Availability

Data available on request due to restrictions eg privacy or ethical. The data presented in this study are available on request from the corresponding author.

## Supplementary Materials

The following are available online at https://www.mdpi.com/article/10.3390/ijms22147462/s1.
