# Supplementary material for: Oxidative Stress and Inflammatory Biomarkers for the Prediction of Severity and ICU Admission in Unselected Patients Hospitalized with COVID-19
Source: Int J Mol Sci. 2021 Jul 12;22(14):7462. doi: 10.3390/ijms22147462 (PMC8306526; doi:10.3390/ijms22147462)
Supplement: Supplementary file 1 [file ijms-22-07462-s001.zip › ijms-1282626-supplementary.pdf]

Oxidative stress and inflammatory biomarkers for the prediction of severity and ICU admission in unselected patients hospitalized with COVID-19"

Supplementary tables

Table S1. Correlations coefficients between studied and routine biomarkers

Type of biomarker	Oxidative Stress Biomarkers			Inflammatory biomarkers					Cell activation biomarkers		
**Biomarker**	**Thiols (log)**	**AOPP (log)**	**IMA (log)**	**IL-6 (log)**	**PSEP (log)**	**CRP (log)**	**Albumin**	**Fibrinogen**	**Calprotectin (log)**	**Leukocytes (log)**	**Neutrophiles (log)**
**Thiols (log)**		-0.313**	-0.506**	-0.460**	-0.438**	-0.445**	0.615**	-0.292**	-0.508**	-0.411**	-0.495**
**AOPP (log)**			0.158*	0.282**	0.172*	0.118	-0.309*	-0.035	0.241*	0.153*	0.249*
**IMA (log)**				0.411**	0.328**	0.580**	-0.357**	0.386**	0.367**	0.248*	0.326**
**IL-6 (log)**					0.546**	0.634**	-0.394**	0.447**	0.463**	0.205*	0.461**
**PSEP (log)**						0.475**	-0.414**	0.281*	0.379**	0.302**	0.429**
**CRP (log)**							-0.458**	0.571**	0.544**	0.250*	0.378**
**Albumin**								-0.199	-0.502**	-0.397**	-0.465**
**Fibrinogen**									0.472**	0.306**	0.357**
**Calprotectin (log)**										0.362**	0.565**
**Leukocytes (log)**											**0.869****
**Neutrophils (log)**											

For this analysis, a log-transformation was performed and indicated by (log) when the distribution was not normal.

* p<0.05; ** p<0.001; Correlation coefficient absolute values below 0.3 are considered to be weak (yellow), from 0.3 to 0.7 are moderate (orange) and above >0.7 are strong (red).

Table S2. Oxidative stress (OS) and inflammation biomarkers concentrations according to increased O_2 needs

Type of biomarker	Biomarker	No increased O_2 needs			Increased O_2 needs			
		N	Median	IQR	N	Median	IQR	***p***
**Oxidative Stress Biomarkers**	**Thiols (µM)**	71	197	149 to 275	89	130	91 to 164	*<0.001*
	**AOPP (µM)**	71	59	7 to 174	88	111	47 to 220	*0.009*
	**IMA (ABSU)**	70	0.17	0.11 to 0.24	88	0.20	0.17 to 0.27	*<0.001*
**Inflammatory biomarkers**	**IL-6 (ng/L)**	71	12.7	1.5 to 32.9	89	80.0	27.8 to 158.8	*<0.001*
	**PSEP (ng/L)**	71	416	250 to 713	88	879	532 to 1668	*<0.001*
**cell activation**	**Calprotectin (mg/L)**	68	11.9	5.0 to 19.3	84	30.4	18.0 to 42.6	*<0.001*

IQR: interquartile range

Table S3. Univariate and multivariate analysis for the prediction of ICU admission and mortality

Baseline characteristics	Prediction of ICU admission				Prediction of mortality			
	Univariate analysis		Multivariate analysis		Univariate analysis		Multivariate analysis	
	**OR [95%CI]**	***p***	**OR [95%CI]**	***p***	**OR [95%CI]**	***p***	**OR [95%CI]**	***p***
**Men –n (%)**	2.20 [1.13-4.27]	*0.019*	/	/	4.28 [1.19 – 15.44]	*0.018*	/	/
**Age >60 years**	1.04 [0.55-1.96]	*0.898*			8.84 [1.96 – 39.9]	*0.001*	16.4 [1.90 – 141]	*0.011*
**Smoking –n (%)**	0.91 [0.42-1.98]	*0.824*			2.81 [0.99 – 7.94]	*0.047*	/	/
**Cardiovascular Disease**	1.86 [0.96-3.59]	*0.065*	/	/	2.10 [0.73 – 6.00]	*0.162*	/	/
**Overweight/obesity**	3.25 [1.40-7.52]	*0.005*	2.83 [1.19-6.74]	*0.019*	1.55 [0.57 – 4.23]	*0.391*		
**Hypertension**	2.00 [1.03-3.87]	*0.039*	/	/	2.20 [0.82 – 5.93]	*0.114*	/	/
**Diabetes**	1.94 [0.92-4.09]	*0.081*	/	/	1.66 [0.58 – 4.78]	*0.347*		
**Chronic kidney Disease**	1.26 [0.44-3.58]	*0.663*			1.10 [0.23 – 5.29]	*0.907*		
**Chronic Resp Failure**	0.93 [0.21-4.04]	*0.924*			2.34 [0.45 – 12.25]	*0.303*		
**Systemic autoimmune Disease**	0.76 [0.18-3.16]	*0.704*			2.32 [0.44 – 12.1]	*0.308*		
**Temperature >38°C**	1.65 [0.78-3.50]	*0.190*	/	/	1.35 [0.41 – 4.39]	*0.615*	/	/
**Caught**	0.77 [0.40-1.48]	*0.440*			0.52 [0.19 – 1.43]	*0.195*	/	/
**Dyspnea**	6.69 [3.04-14.7]	*<0.001*	3.67 [1.45-9.28]	*0.006*	2.30 [0.71 – 7.41]	*0.155*	/	/

**Myalgias**	0.49 [0.24-1.01]	*0.052*	/	/	0.27 [0.06 – 1.23]	*0.076*	/	/
**Fatigue**	0.68 [0.35-1.31]	*0.246*			0.79 [0.18 – 2.26]	*0.668*		
**Diarrhea**	0.56 [0.26-1.21]	*0.141*	/	/	0.36 [0..08 – 1.65]	*0.171*	/	/
**Oxygenation**	39.5 [5.2-297.2]	*<0.001*	/	/	6.64 [0.86 – 51.46]	*0.012*	/	/
**Thiols values (log-transformed)**	0.01 [0.00-0.05]	*<0.001*	0.05 [0.01-0.58]	0.017	0.03 [0.00 – 0.25]	0.001	/	/
**IL-6 values (lor-transformed)**	4.0 [2.28-6,98]	*<0.001*	2.56 [1.20-5.47]	*0.015*	2.50 [1.31 – 4.78]	*0.006*	/	/
**Calprotectin values (log-transformed)**	10.48 [3.9-28.4]	*<0.001*	/	/	19.1 [3.23 – 112.9]	*0.001*	33.7 [3.37 - 337]	*0.003*

OR, odds ratio. Grey zones indicate variables not included in the multivariate analysis.

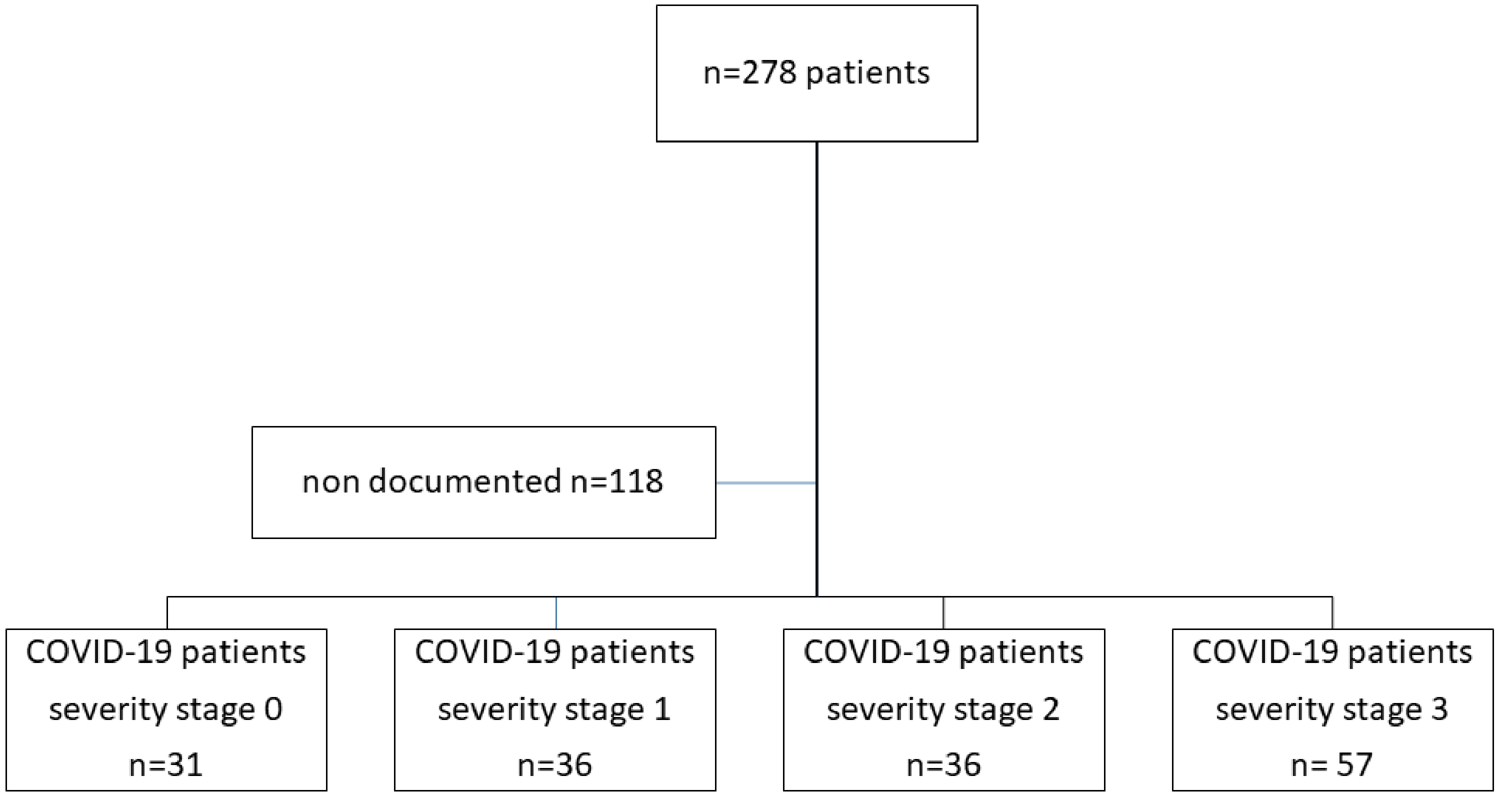
n=278 patients
non documented n=118
COVID-19 patients
severity stage 0
n=31
COVID-19 patients
severity stage 1
n=36
COVID-19 patients
severity stage 2
n=36
COVID-19 patients
severity stage 3
n= 57

Figure S1

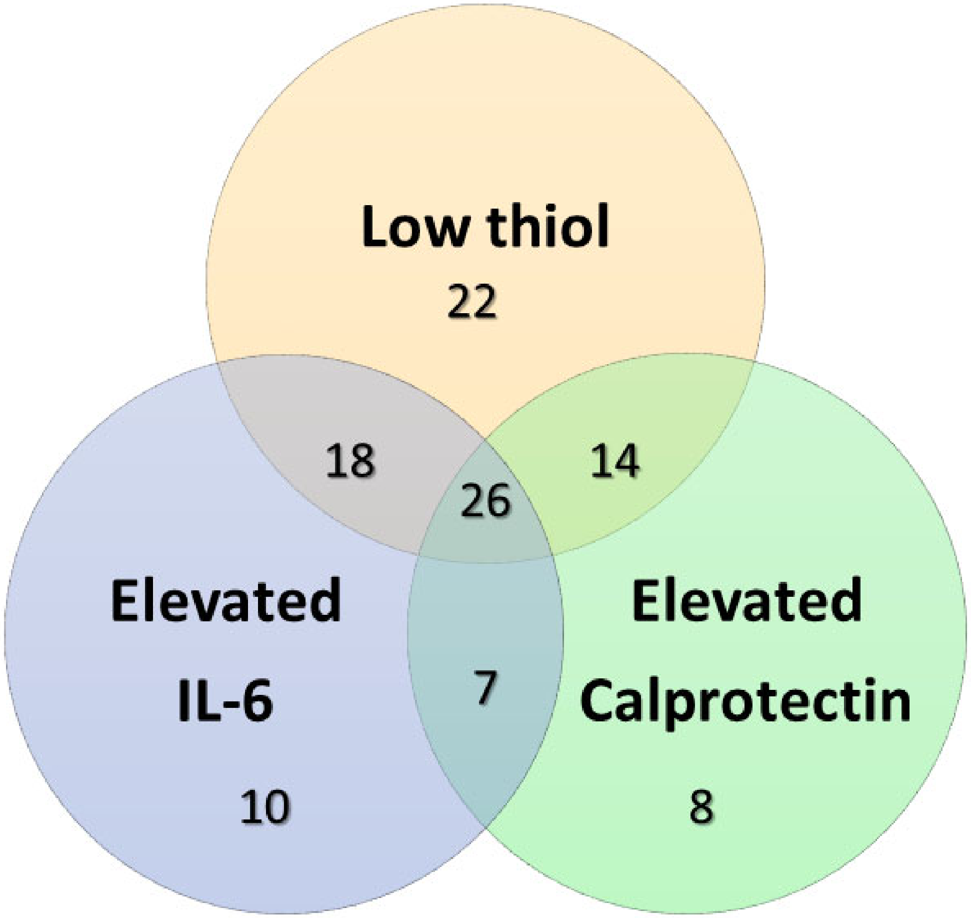
Low thiol
22
18
14
26
Elevated
IL-6
7
Elevated
Calprotectin
10
8

Figure S2
